# Resveratrol Inhibits Growth of Orthotopic Pancreatic Tumors through Activation of FOXO Transcription Factors

**DOI:** 10.1371/journal.pone.0025166

**Published:** 2011-09-27

**Authors:** Sanjit K. Roy, Qinghe Chen, Junsheng Fu, Sharmila Shankar, Rakesh K. Srivastava

**Affiliations:** 1 Department of Pharmacology, Toxicology and Therapeutics, and Medicine, The University of Kansas Cancer Center, The University of Kansas Medical Center, Kansas City, Kansas, United States of America; 2 Department of Pathology and Laboratory Medicine, The University of Kansas Cancer Center, The University of Kansas Medical Center, Kansas City, Kansas, United States of America; Roswell Park Cancer Institute, United States of America

## Abstract

**Background:**

The forkhead transcription factors of the O class (FOXO) play a direct role in cellular proliferation, oxidative stress response, and tumorigenesis. The objectives of this study were to examine whether FOXOs regulate antitumor activities of resveratrol in pancreatic cancer cells *in vitro* and *in vivo*.

**Methodology/Principal Findings:**

Pancreatic cancer cell lines were treated with resveratrol. Cell viability, colony formation, apoptosis and cell cycle were measured by XTT, soft agar, TUNEL and flow cytometry assays, respectively. FOXO nuclear translocation, DNA binding and transcriptional activities were measured by fluorescence technique, gelshift and luciferase assay, respectively. Mice were orthotopically implanted with PANC1 cells and orally gavaged with resveratrol. The components of PI3K and ERK pathways, FOXOs and their target gene expressions were measured by the Western blot analysis. Resveratrol inhibited cell viability and colony formations, and induced apoptosis through caspase-3 activation in four pancreatic cancer cell lines (PANC-1, MIA PaCa-2, Hs766T, and AsPC-1). Resveratrol induced cell cycle arrest by up-regulating the expression of p21/CIP1, p27/KIP1 and inhibiting the expression of cyclin D1. Resveratrol induced apoptosis by up-regulating Bim and activating caspase-3. Resveratrol inhibited phosphorylation of FOXOs, and enhanced their nuclear translocation, FOXO-DNA binding and transcriptional activities. The inhibition of PI3K/AKT and MEK/ERK pathways induced FOXO transcriptional activity and apoptosis. Furthermore, deletion of FOXO genes abrogated resveratrol-induced cell cycle arrest and apoptosis. Finally, resveratrol-treated mice showed significant inhibition in tumor growth which was associated with reduced phosphorylation of ERK, PI3K, AKT, FOXO1 and FOXO3a, and induction of apoptosis and FOXO target genes.

**Conclusions:**

These data suggest that inhibition of ERK and AKT pathways act together to activate FOXO transcription factors which are involved in resveratrol-mediated pancreatic tumor growth suppression.

## Introduction

Cancer of the pancreas is the fourth leading cause of cancer-related mortality in the United States with a 5-year survival less than 5% [1]. Pancreatic ductal adenocarcinoma is a highly aggressive disease that invariably evades early diagnosis [2]. The mean survival time for patients with metastatic disease is only 3–6 months, and only 20–30% of pancreatic cancer cases are alive after 12 months. Several factors are associated with increased risk for pancreatic cancer and these include chronic pancreatitis, smoking, diabetes, prior gastric surgery, exposure to certain classes of organic solvents, radiation, and specific gene polymorphisms [3], [4]. Heritable as well as several acquired gene mutations have been identified in pancreatic tumors [5]. The K-*Ras* oncogene is primarily mutated in codon 12 in >90% of pancreatic tumors and the mutation results in a constitutively active form of ras that can lead to increased cell proliferation. Mutations in the tumor suppressor gene *p53*, the cyclin-dependent kinase inhibitor p16, and SMAD4, a downstream target of TGFβ also exhibit high mutation frequencies in pancreatic tumors.

Since pancreatic cancer is frequently detected at an advanced stage, it has limited treatment options and insignificant improvements in tumor regression and overall survival [6]. 5-Fluorouracil alone or in combination with other drugs has been extensively used for treatment of advanced pancreatic cancer, and gemcitabine, a deoxycytidine analog (or antimetabolite), has partially replaced 5-fluorouracil as a treatment for pancreatic cancer. Therefore, effective strategies are urgently needed to prevent pancreatic cancer. Resveratrol is a polyphenolic phytochemical that exhibits a broad spectrum of anticancer activities against multiple tumor types, including pancreatic cancer [7]. In vitro, resveratrol inhibited the proliferation of human pancreatic cancer cell lines, synergized the apoptotic effects of gemcitabine, and inhibited the constitutive activation of NFκB and its gene products [8]. *In vivo*, resveratrol significantly suppressed the growth of the tumor and this effect was further enhanced by gemcitabine [8]. Resveratrol also suppressed the NFκB activation and expression of cyclin D1, COX-2, ICAM-1, MMP-9 and survivin in tumor tissues [8]. Furthermore, we recently demonstrated that resveratrol induced apoptosis by activating capase-3/7 and inhibiting the expression of Bcl-2 and XIAP in human pancreatic cancer stem cells (CSCs) [9]. Resveratrol inhibited pluripotency maintaining factors (Nanog, Sox-2, c-Myc and Oct-4) and drug resistance gene ABCG2 in CSCs. Inhibition of Nanog by shRNA enhanced the inhibitory effects of resveratrol on self-renewal capacity of CSCs. Finally, resveratrol inhibited CSC's migration and invasion and markers of epithelial-mesenchymal transition (EMT) such as Zeb-1, Slug and Snail. Overall, resveratrol inhibited pancreatic CSC characteristics in human and Kras^G12D^ transgenic mice by inhibiting pluripotency maintaining factors and EMT.

Transcription factors of the Forkhead box O (FOXO) class are predominantly regulated through phosphoinositide 3-kinase/AKT (also known as PKB) pathway. FOXO1a/FKHR, FOXO3a/FKHRL1, and FOXO4/AFX are members of FOXO subfamily [10]. The PI3K/AKT pathway phosphorylates all of these FOXO proteins, resulting in impairment of DNA binding ability and inhibition of FOXO-dependent transcription [11]. Inhibition of the PI3K/AKT pathway leads to dephosphorylation and nuclear translocation of active FOXOs, which causes cell cycle arrest and apoptosis [12]. Conversely, loss of PTEN activity results in increased AKT activity leading to inhibition of FOXO activity through phosphorylation and cytoplasmic sequestration [12]. FOXO transcriptional activity controls cellular proliferation and apoptosis downstream of PTEN, PI3K and AKT [13]. Since inactivation and loss of PTEN, and overexpression of AKT are frequently observed in pancreatic cancer [14], targeting a downstream target such as FOXO may be an attractive idea for pancreatic cancer prevention and/or treatment.

In addition to PI3K/AKT, MAP kinase pathway has also been shown to regulate FOXO activities. The Ras proteins transduce signals from ligand-activated tyrosine kinase receptors to downstream effectors which can lead to cellular transformation [15], [16]. Constitutive activation of the Ras-ERK/MAPK signaling pathway often leads to promotion of abnormal cell growth and tumorigenesis [17]. In mammalian cells, ERK [18], p38 MAP kinase [19], and JNK [20], [21] regulate mitosis, movement, metabolism and apoptosis. These MAP kinases are activated by the dual phosphorylations of neighboring threonine and tyrosine residues in response to various extracellular stimuli [22]. Specifically, ERKs are often activated by growth signals [23], whereas p38 and JNK are activated by stress-responsive signal [20]. Activation of ERK leads to phosphorylation and inactivation of FOXO proteins. Since FOXO proteins can be phosphorylated by AKT and ERK, inhibition of these pathways could be considered as a novel strategy for the prevention and/or treatment of pancreatic cancer. In support of this hypothesis, we have recently demonstrated that inhibition of PI3K/AKT and MEK/ERK pathways act synergistically to regulate anti-angiogenic effects of EGCG and sulforaphane through activation of FOXO transcription factors and downstream target genes [24], [25].

Although resveratrol has been shown to inhibit pancreatic cancer cell growth, its effects on the regulation of FOXO transcription factors and downstream targets in pancreatic cancer have never been examined. Furthermore, resveratrol, which simultaneously inhibits the activation of PI3K/AKT and MEK/ERK pathways (resulting in activation of FOXO), could be an attractive candidate for the management of pancreatic cancer. The objectives of our study were to examine the molecular mechanisms by which resveratrol inhibits pancreatic cancer growth in vitro and in vivo. Our results demonstrate that resveratrol induced the expression of PTEN, phosphorylation of JNK and p38, and inhibited phosphorylation of PI3K, AKT, ERK, FOXO1 and FOXO3a leading to growth arrest and apoptosis. Furthermore, inhibition of FOXOs by shRNA blocked anti-proliferative and proapoptotic effects of resveratrol. Finally, resveratrol-treated mice showed significant inhibition in tumor growth which was associated with reduced phosphorylation of ERK, PI3K, AKT, FOXO1 and FOXO3a, and induction of apoptosis and FOXO target genes in tumor tissues.

## Results

### Inhibition of pancreatic cancer cell viability and colony formation by resveratrol

**Figure 1:**
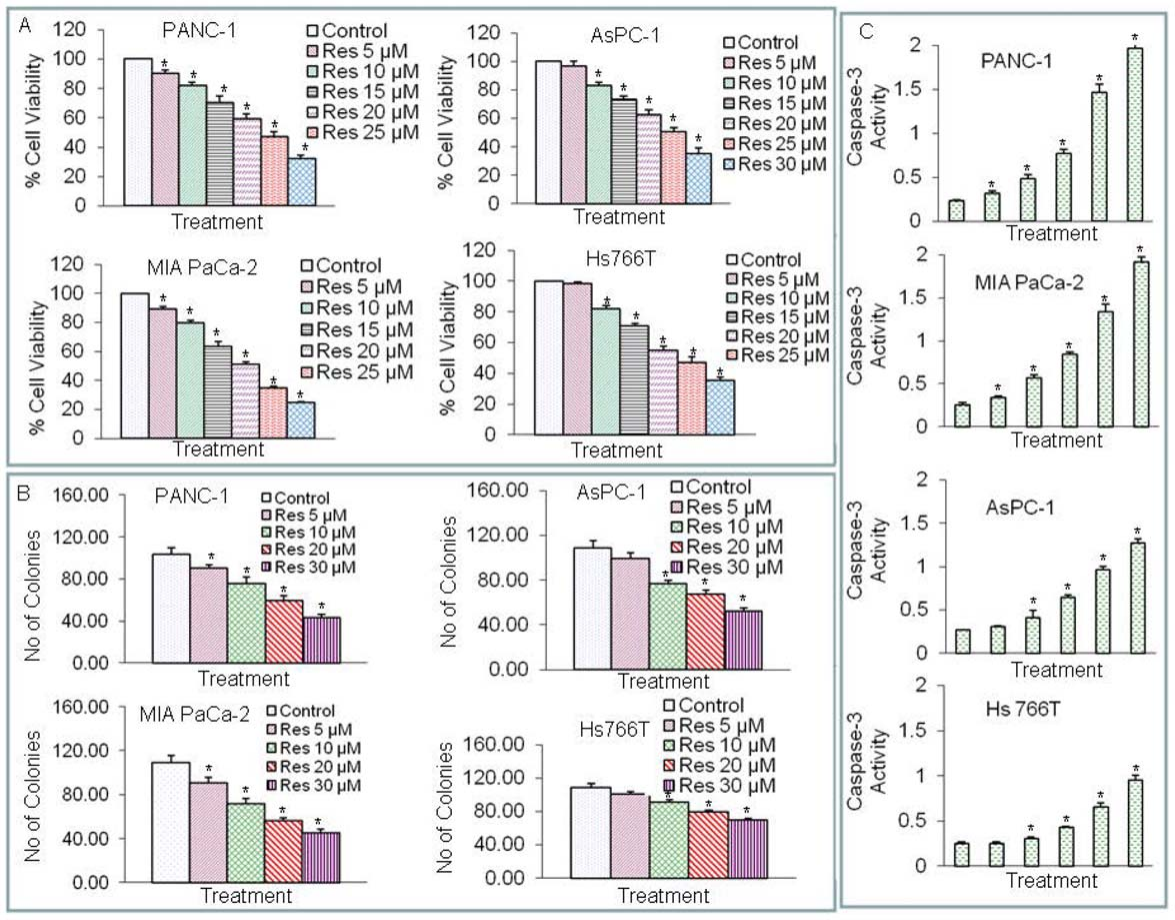
Effects of resveratrol on cell viability, colony formation and caspase-3 activity in pancreatic cancer cells. (A), Cell Viability. Pancreatic cancer (PANC-1, MIA PaCa-2, Hs766T and AsPC-1) cells were treated with resveratrol (0–30 µM) for 48 h. Cell viability was measured by XTT assay. Data represent the mean ± S.D. * = significantly different from respective controls, P<0.05. (B), Colony formation. Pancreatic cancer (PANC-1, MIA PaCa-2, Hs766T and AsPC-1) cells were treated with resveratrol (0–30 µM) for 3 weeks. At the end of incubation period, colonies were fixed and number of colonies were counted. Data represent the mean ± S.D. * = significantly different from respective controls, P<0.05. (C), Caspase-3 activity. Pancreatic cancer PANC-1, MIA PaCa-2, Hs 766T and AsPC-1 cells were treated with resveratrol (0–40 µM) for 24 h and caspase-3 activity was measured as per manufacturer's instructions (EMD Biosciences). Data represent the mean ± S.D. * = significantly different from respective controls, P<0.05.

We first examined the effects of resveratrol on cell viability of four pancreatic cancer cell lines by XTT assay. These four pancreatic cancer cell lines (MIA PaCa-2, AsPC-1, PANC-1 and Hs766T) were selected due to their different genetic background and pathological stages. As shown in Fig. 1A, resveratrol inhibited cell viability in a dose-dependent manner. PANC-1 and MIA PaCa-2 cell lines were most sensitive, AsPC-1 cell line was moderately sensitive, and Hs 766T cell line was least sensitive. These data demonstrate the anti-proliferative effects of resveratrol on human pancreatic cancer cells.

We next examined the effects of resveratrol on colony formation on four pancreatic cancer cell lines by soft agar assay. Resveratrol inhibited colony formation in a dose-dependent manner (Fig. 1B). Colonies formed by PANC-1 and MIA PaCa-2 cells were most sensitive, AsPC-1 cell line was moderately sensitive, and Hs 766T cell line was least sensitive. These data suggest that resveratrol can be used as a potent chemopreventive/therapeutic agent for pancreatic cancer.

Mitochondria play a major role in apoptosis which can be induced by active caspase-3 [26]. We therefore examined whether resveratrol induces apoptosis through activation of caspase-3 in pancreatic cancer cell lines (Fig. 1C). Resveratrol induced caspase-3 activity in PANC-1, MIA PaCa-2, Hs 766T and AsPC-1 cells. However, the activation of caspase-3 was less in Hs766T cells compared to other pancreatic cancer cell lines. These data suggest that resveratrol induces apoptosis through activation of caspase-3 in pancreatic cancer cells.

### Regulation of apoptosis and cell cycle proteins in pancreatic cancer cells by resveratrol

**Figure 2:**
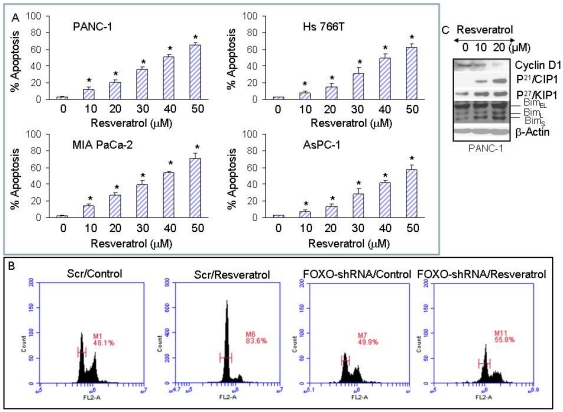
Effect of resveratrol on apoptosis and cell cycle. (A), Pancreatic cancer PANC-1, MIA PaCa-2, Hs 766T and AsPC-1 cells were treated with resveratrol (0–30 µM) for 48 h and apoptosis was measured by TUNEL assay. Data represent the mean ± S.D. * = significantly different from respective controls, P<0.05. (B), PANC-1 cells were transfected with either scrambled or FOXO1/FOXO3a/FOXO4 shRNA expressing constructs and treated with resveratrol (10 µM) for 24 h. At the end of incubation period, cells were harvested, and cell cycle analysis was performed by flow cytometry. (C), Effects of resveratrol on cell cycle regulatory genes. PANC-1 cells were treated with resveratrol (0–20 µM) for 24 h. The expression of cyclin D1, p21^/CIP1^, p27^/KIP1^ and Bim was measured by Western blotting. Anti β-actin antibody was used as a loading control.

We next examined the effects of resveratrol on apoptosis (Fig. 2A). Resveratrol induced apoptosis in PANC-1, MIA PaCa-2, HS766T and AsPC-1 cells in a dose-dependent manner. PANC-1 and MIA PaCa-2 cell lines were most sensitive, AsPC-1 cell line was moderately sensitive, and Hs 766T cell line was least sensitive. These data suggest that resveratrol can induce apoptosis in pancreatic cancer cells irrespective of their Kras status, and thus can be used as a cancer preventive agent.

PI3K/AKT signaling pathway has been shown to regulate cell cycle progression through regulation of FOXO transcription factors [27]. We therefore examined the effects of FOXO transcription factors on resveratrol-induced cell cycle arrest by inhibiting the FOXO transcription factors by shRNA (Fig. 2B). FOXO1/FOXO3a/FOXO4 shRNA expression vectors have been described elsewhere [28]. Treatment of PANC-1/scrambled cells with resveratrol resulted in cell cycle arrest at G1 stage (Control = 48.1%, resveratrol = 83.6%). By comparison, inhibition of FOXO1/FOXO3a/FOXO4 by shRNA blocked resveratrol-induced growth arrest. These data suggest that FOXO transcription factors are required for resveratrol-induced cell cycle arrest. We next examined the effects of resveratrol on FOXO target genes (Fig. 2C). Resveratrol inhibited the expression of cyclin D1 and induced the expression of cell cycle inhibitors (p21/CIP1 and p27/KIP1) and Bim in PANC-1 cells. Over all, these data suggest that resveratrol causes growth arrest and induces apoptosis through regulation of FOXO target genes such as cyclin D1, p21/CIP1, p27/KIP1 and Bim.

### Inhibition of PI3K/AKT and MAP kinase pathways enhance resveratrol-induced apoptosis

**Figure 3:**
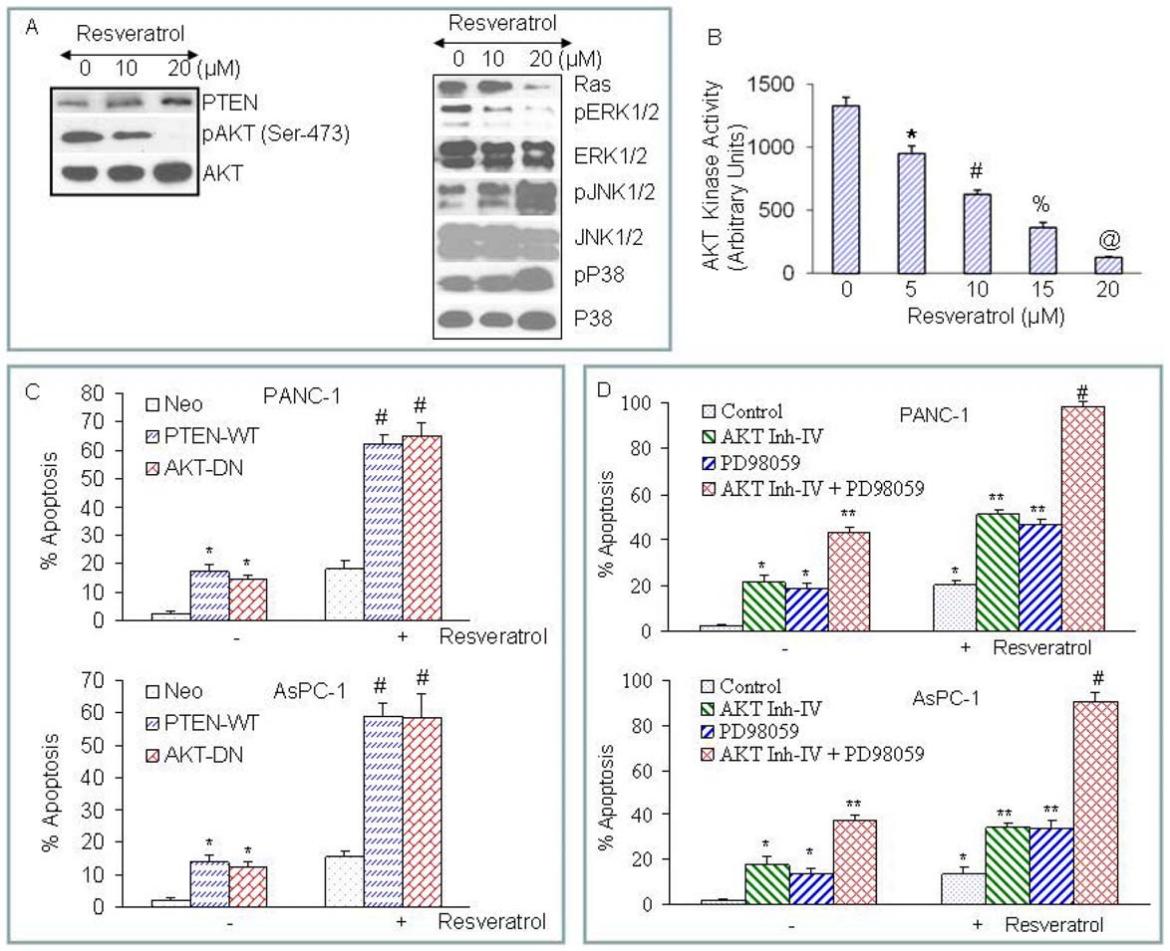
Effects of resveratrol on the expression of PTEN, AKT and MAP kinases; and the effects of PI3K/AKT and MAPK pathways on resveratrol-induced apoptosis. (A), PANC-1 cells were treated with or without resveratrol (0–20 µM) for 24 h. The cells were harvested and the expression of PTEN, phospho-AKT, AKT, Ras, phospho-ERK, ERK, phospho-JNK, JNK, phospho-p38 and p38 was measured by Western blotting. (B), AKT kinase activity. PANC-1 cells were treated with resveratrol (0–20 µM) for 24 h. AKT kinase activity was measured as we described in materials and methods. Data represent the mean ± S.D. *, #, %, and @ = significantly different from respective controls, P<0.05. (C), PTEN and dominant negative AKT enhance resveratrol-induced apoptosis. PANC-1 and AsPC-1 cells were transiently transfected with empty vector (pcDNA3.1), PTEN wild type (PTEN-WT) or dominant negative AKT (AKT-DN) along with pCMV-LacZ vector (as transfection control) for 24 h. After medium replacement, cells were treated with resveratrol (10 µM) for 48 h and, apoptosis was measured by TUNEL assay. Data represent the mean ± S.D. * and # = significantly different from respective controls, P<0.05. (D), PANC-1 and AsPC-1 cells were pretreated with AKT inhibitor IV (1 µM) and/or MEK1/2 inhibitor PD98059 (10 µM) for 2 h, followed by treatment with or without resveratrol (10 µM) for 48 h. At the end of incubation period, cells were harvested and apoptosis was measured by TUNEL assay. Data represent mean ± SD. *, ** and # = significantly different from respective controls, P<0.05.

The constitutively AKT has been shown to enhance survival of cancer cells and also to be the cause of drug-resistance [29]. Since PTEN regulates AKT activity, we measured the expression of PTEN and phosphorylation status of AKT in pancreatic cancer cells treated with resveratrol (Fig. 3A). Resveratrol induces PTEN expression and inhibits AKT phosphorylation in PANC-1 cells. By comparison, resveratrol has no effect on total AKT expression. We next confirmed the inhibition of AKT activation by resveratrol with kinase assay (Fig. 3B). Resveratrol inhibited AKT kinase activity in a dose-dependent manner. These data suggest that resveratrol inhibits cell proliferation by regulating PI3K/AKT pathway.

Ras/Raf/MAP kinase pathway has been shown to regulate migration, mitosis, metabolism, and apoptosis [20]. We therefore examined the effects of resveratrol on the expression of Ras, and activation of ERK, JNK and p38 MAP kinases. Resveratrol inhibited Ras expression in PANC-1 cells (Fig. 3A). Treatment of PANC-1 cells with resveratrol caused a decrease in ERK phosphorylation, and an increase in JNK phosphorylation. By comparison, treatment of PANC-1 cells with resveratrol resulted in a slight increase in the phosphorylation of p38 MAP kinase. These data suggest that resveratrol inhibits growth and induces apoptosis through regulation of Ras/Raf/MAP kinase pathway.

PI3K/AKT pathway has been shown to inhibit apoptosis. We next examined whether resveratrol induces apoptosis through PI3K/AKT pathway (Fig. 3C). Pancreatic cancer cells were transfected with empty vector, wild type PTEN, dominant negative AKT (DN-AKT), and apoptosis was measured. Overexpression of wild type PTEN or DN-AKT induced apoptosis in AsPC-1 and PANC-1 cells. Treatment of these transfected cells with resveratrol further enhanced apoptosis. These data suggest that inhibition of PI3K/AKT pathway enhances resveratrol-induced apoptosis in pancreatic cancer cells.

Since inhibition of PI3K/AKT and MEK/ERK pathways together induce apoptosis in an additive or synergistic manner, we sought to examine whether these pathways act together to regulate resveratrol-induced apoptosis. The activations of AKT and MEK/ERK were inhibited by AKT Inh-IV and PD98059, respectively. AKT inhibitor (AKT Inh-IV) and MEK1/2 inhibitor (PD98059) alone induced apoptosis in both PANC-1 and AsPC-1 cells (Fig. 3D). AKT inhibitor and MEK1/2 inhibitor (PD98059) synergistically/additively induced apoptosis in PANC-1 and AsPC-1 cells. Interestingly, the combination of AKT inhibitor and PD98059 with resveratrol induced more apoptosis than AKT inhibitor plus resveratrol or PD98059 plus resveratrol. Based on these data, it can be suggested that the inhibition of PI3K/AKT and MEK/ERK pathways act together to enhance resveratrol-induced apoptosis.

### FOXO transcription factors regulate resveratrol-induced apoptosis in pancreatic cancer cells

**Figure 4:**
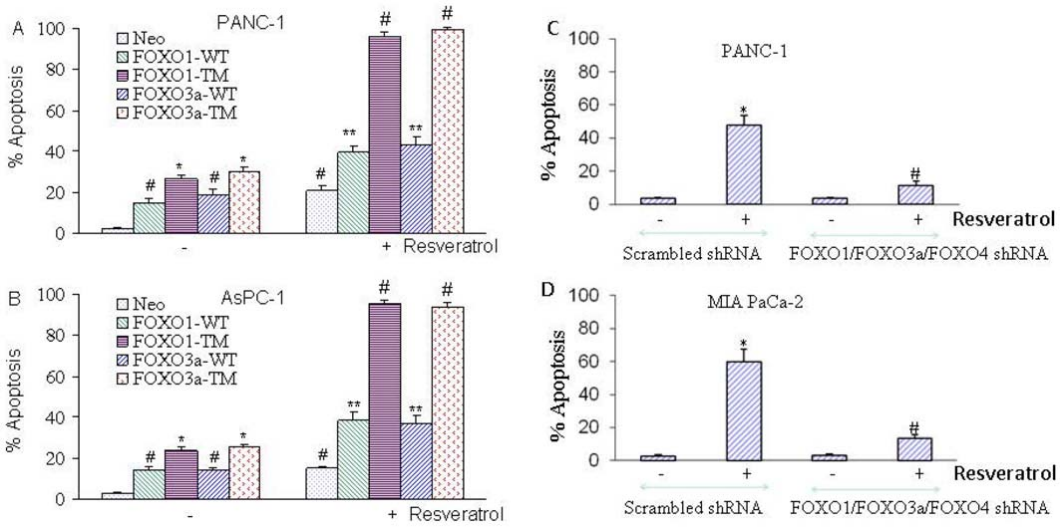
Effects of FOXO transcription factors on resveratrol induced apoptosis. (A and B), Phosphorylation deficient mutants of FOXO enhance resveratrol-induced apoptosis in pancreatic cancer cells. PANC-1 and AsPC-1 cells were transiently transfected with empty vector or plasmids encoding FOXO1-TM, FOXO3a-TM, or FOXO4-TM for 24 h. After transfection, culture medium was replaced with complete RPMI medium and treated with resveratrol (10 µM) for 48 h. Apoptosis was measured by TUNEL assay. Data represent the mean ± S.D. *, #, **,  = significantly different from control, P<0.05. (C and D), PANC-1 and MIA PaCa-2 cells were stably transfected with either scrambled or FOXO1, FOXO3a and FOXO4 shRNA plasmids. Transfected cells were treated with resveratrol (10 µM) and apoptosis was measured by TUNEL assay. Data represent the mean ± S.D. *, # = significantly different from respective controls, P<0.05.

Inhibition of PI3K/AKT and MEK/ERK pathways activates FOXO transcription factors. We therefore examined whether FOXO transcription factors regulate apoptosis induced by resveratrol in pancreatic cancer cells. PANC-1 and AsPC-1 cells were transfected with FOXO1-WT, FOXO1-TM (phosphorylation mutant), FOXO3a or FOXO3a-TM (phosphorylation deficient mutant) (Fig. 4A and B). Wild type and phosphorylation deficient mutants of FOXO expression plasmids have previously been described [24]. Overexpression of FOXO1, FOXO1-TM, FOXO3a and FOXO3a-TM induced apoptosis in both PANC-1 and AsPC-1 cells. However, phosphorylation deficient mutants of FOXO1 and FOXO3a induced more apoptosis than wild type FOXO1 and FOXO3a. Resveratrol also induced apoptosis in both PANC-1 and AsPC-1 cells. Overexpression of FOXO1-WT, FOXO1-TM, FOXO3a or FOXO3a-TM further enhanced resveratrol-induced apoptosis. Phosphorylation deficient mutants of FOXO1 and FOXO3a had more effects on resveratrol-induced apoptosis than wild type counterparts. These data suggest that the phosphorylation of FOXO transcription factors plays a major role in their activities, and FOXO (wild type and triple mutant) can enhance resveratrol-induced apoptosis.

We next examined whether inhibition of FOXO transcription factors by shRNA abolishes resveratrol-induced apoptosis (Fig. 4C and D). Pancreatic cancer PANC-1 and MIA PaCa-2 cells were stably transfected with plasmids expressing FOXO1, FOXO3a and FOXO4 shRNA. These expression vectors have been described elsewhere [28]. Resveratrol induced apoptosis in PANC-1 and MIA PaCa-2 cells transfected with scrambled vectors. By comparison, inhibition of FOXO1, FOXO3a and FOXO4 through shRNA inhibited resveratrol induced apoptosis in PANC-1 and MIA PaCa-2 cells. These data suggest that FOXO transcription factors are required, at least in part, for induction of apoptosis by resveratrol.

### Inhibition of PI3K/AKT and MEK/ERK pathways or overexpression of FOXO transcription factor synergistically induces FOXO transcriptional activity in the presence or absence of resveratrol

**Figure 5:**
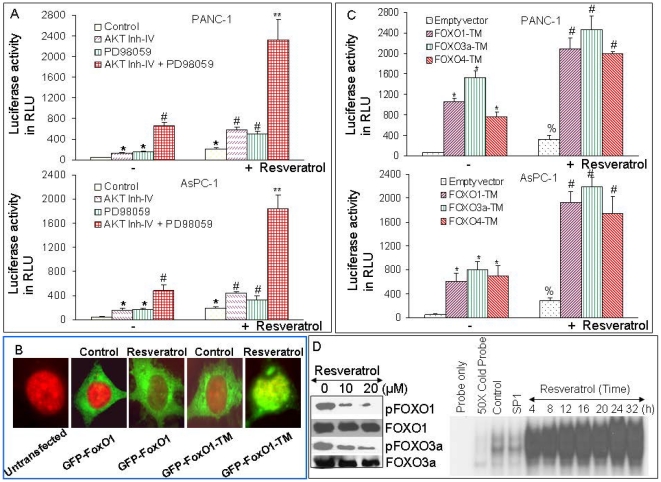
Inhibition of PI3K/AKT and MEK/ERK pathways synergistically/additively enhanced resveratrol -induced apoptosis and FOXO transcriptional activity in pancreatic cancer cells. (A), PANC-1 and AsPC-1 cells were transiently transfected with 6× DBE-luciferase construct for 24 h. After transfection, cells were pretreated with AKT inhibitor IV (1 µM) and/or MEK1/2 inhibitor PD98059 (10 µM) for 2 h, followed by treatment with or without resveratrol (10 µM) for 24 h. Cells were harvested for firefly/Renilla luciferase assays using the Dual-Luciferase Reporter Assay System (Promega). Luciferase counts were normalized using *Renilla* luciferase transfection control (pRL-TK; Promega). Data represent the mean ± S.D. *, # and ** = significantly different from respective controls, P<0.05. (B), Resveratrol induces translocation of wild type and triple mutant FOXO1 to nucleus. Pancreatic cancer PANC-1 cells were transfected with either GFP-FOXO1 or GFP-FOXO-TM and treated with resveratrol (10 µM) for 24 h. Cells were visualized by fluorescence microscopy. (C), Phosphorylation deficient mutants of FOXO (triple mutants) enhance resveratrol-induced FOXO transcriptional activity in pancreatic cancer. PANC-1 and AsPC-1 cells were transiently transfected with empty vector or constructs encoding FOXO1-TM, FOXO3a-TM, or FOXO4-TM together with 6× DBE-luciferase for 24 h. After transfection, culture medium was replaced with complete RPMI, treated with resveratrol (10 µM) for 24 h, and harvested for firefly/Renilla luciferase assays using the Dual-Luciferase Reporter Assay System (Promega). Luciferase counts were normalized using *Renilla* luciferase transfection control (pRL-TK; Promega). Data represent the mean ± S.D. *, % and # = significantly different from control, P<0.05. (D) Left panel, Resveratrol inhibits cytoplasmic phosphorylation of FOXO1 and FOXO3a. PANC-1 cells were treated with resveratrol (0, 10 and 20 µM) for 36 h, and cytoplasmic fractions were prepared. The expressions of p-FOXO1 and p-FOXO3a were measured by the Western blot analysis. Right Panel, FOXO-DNA binding. PANC-1 cells were treated with or without resveratrol (10 µM) for various time points (0–32 h). Nuclear extracts were prepared. Gelshift assay was performed as we described in Material and Methods. Lane 1 = probe only, lane 2 = 50× cold probe, lane 3 = control, lane 4 = cell lysates from control samples treated with SP1 probe, lanes 5–11 = cell lysate from resveratrol treated samples at 4, 8, 12, 16, 20, 24 and 32 h.

Since inhibition of PI3K/AKT and MEK/ERK pathways synergistically induces apoptosis in pancreatic cancer cells, we next sought to examine whether inhibition of these two pathways act together to regulate FOXO activity. FOXO-luciferase construct (pGL3-6X DBE) have previously been described [24]. AKT inhibitor (AKT Inh-IV) and MEK1/2 inhibitor (PD98059) synergistically induced FOXO transcriptional activity in AsPC-1 and PANC-1 cells (Fig. 5A). AKT Inh-IV or PD98059 enhanced resveratrol-induced FOXO transcriptional activity. Interestingly, the combination of AKT Inh-IV and PD98059 with resveratrol-induced higher FOXO transcriptional activity than AKT Inh-IV plus resveratrol or PD98059 plus resveratrol. These data suggest that inhibition of PI3K/AKT and MEK/ERK pathways acts synergistically/additively to regulate FOXO transcriptional activity in the absence or presence of resveratrol.

Since inhibition of FOXO1 (FKHR) transcription factor enhanced resveratrol-induced apoptosis in pancreatic cancer cells, we sought to examine whether resveratrol enhances translocation of wild type and phosphorylation deficient mutant of FOXO1 to nucleus (Fig. 5B). PANC-1 cells were transfected with GFP-FOXO1 or GFP-FOXO1-TM plasmids and treated with resveratrol. The translocation of FOXO1 to nucleus was measured by fluorescence microscopy. Resveratrol induced translocation of FOXO1 to nucleus. However, the translocation of phosphorylation deficient mutant of FOXO1 was much higher than wild type FOXO1. These data suggest that resveratrol can regulate translocation of FOXO transcription factor to the nucleus.

We next examined whether resveratrol induces transcriptional activation of FOXO in the presence or absence phosphorylation deficient triple mutants of FOXO proteins (FOXO1-TM, FOXO3a-TM, or FOXO4-TM) (Fig. 5C). PANC-1 and AsPC-1 cells were transfected with wild type FOXO promoter linked to a luciferase reporter gene in the presence or absence of plasmids expressing FOXO1-TM, FOXO3a-TM, or FOXO4-TM. After transfection, cells were treated with resveratrol for 24 h, and luciferase activity was measured. Transfection of cells with plasmids expressing FOXO1-TM, FOXO3a-TM, or FOXO4-TM induced FOXO transcriptional activity compared with the empty vector (control). Resveratrol also induced FOXO transcriptional activity. Interestingly, resveratrol-induced FOXO transcriptional activity was further enhanced in the presence of FOXO1-TM, FOXO3a-TM, and FOXO4-TM. These data indicate that FOXO transcription factor may play a major role in mediating biological effects of resveratrol in pancreatic cancer cells.

Since resveratrol induces apoptosis and FOXO transcriptional activity, we next sought to examine the effects of resveratrol on phosphorylation of FOXO1 and FOXO3a in the cytoplasmic fractions (Fig. 5D, left Panel). Resveratrol inhibited the phosphorylation of FOXO1 and FOXO3a in PANC-1 cells. Since resveratrol inhibited the phosphorylation of FOXO1 and FOXO3a and enhanced the nuclear translocation of wild type and mutant FOXO1-TM, we next sought to examine the FOXO-DNA binding activity by gelshift assay (Fig. 5D, right panel). Treatment of PANC-1 cells with resveratrol resulted in enhanced FOXO-DNA binding activity reaching maximum at 24 h. The FOXO-DNA binding in the presence of SP1 (lysate from untreated cells) was similar to untreated control. Overall, these data suggest that resveratrol can inhibit the phosphorylation of FOXO transcription factors, leading to their translocation to nucleus and enhanced FOXO transcriptional activity.

### Resveratrol inhibits the expression of Sirt1, Sirt2 and Sirt3 in pancreatic cancer cells

**Figure 6:**
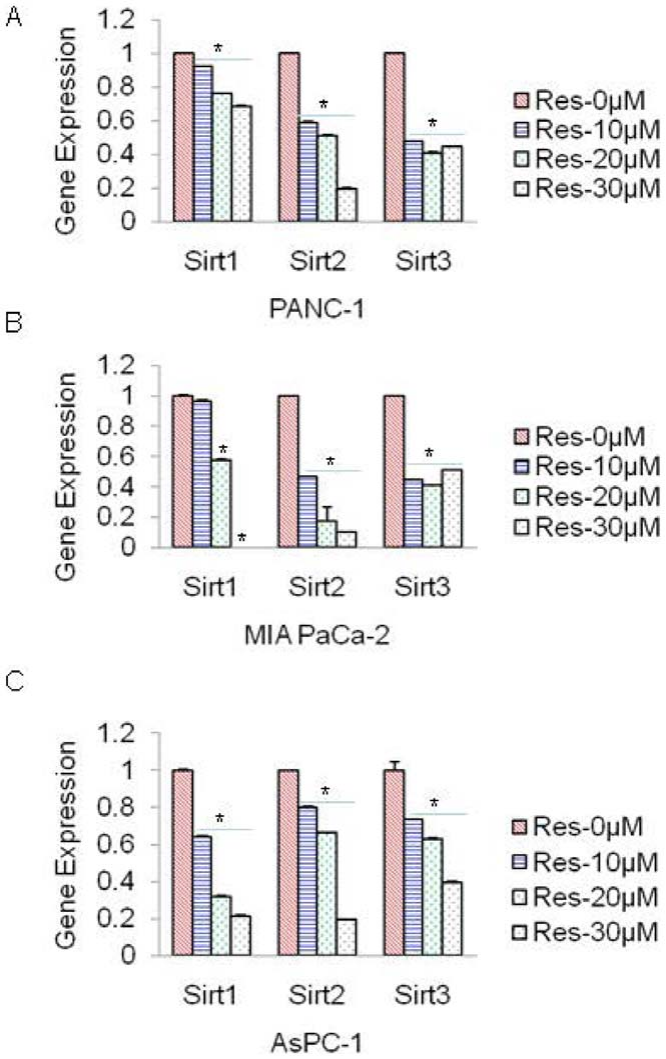
Inhibtion of Sirts by resveratrol in pancreatic cancer cells. (A–C) Pancreatic cancer PANC-1, MIA PaCa-2 and AsPC-1 cells were treated with resveratrol (0–30 µM) for 24 h, and the expression of Sirt1, Sirt2 and Sirt3 was measured by qRT-PCR. Data represent mean ± SD. * = significantly different from respective control, P<0.05.

Since Sirts regulate tumor formation and angiogenesis, we next examined the effects of resveratrol on the expression of Sirt1, Sirt2 and Sirt3 in pancreatic cancer cells by q-RT-PCR. Resveratrol inhibited the expression of Sirt1, Sirt2 and Sirt3 in PANC-1, MIA PaCa-2 and AsPC-1 cells (Fig. 6). These data suggest that anti-proliferative effects of resveratrol may be exerted through Sirts.

### Resveratrol inhibits the growth of PANC-1 cells orthotopically implanted in Balb C Nude mice by inhibiting FOXO transcription factors

**Figure 7:**
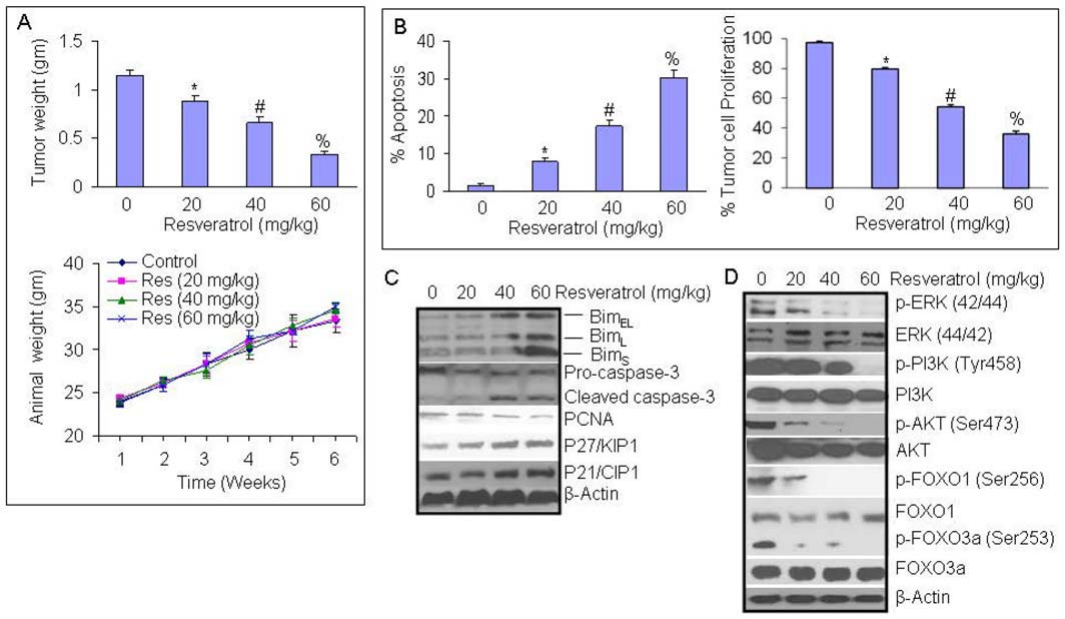
Resveratrol inhibits the growth of PANC-1 tumors orthotopically inplanted in Balb C Nude mice by inhibiting FOXO transcription factors. (A) Upper Panel, PANC-1 cells were orthotopically implanted into the pancreas of Balb C nude mice. Tumor bearing mice were treated with resveratrol (0–60 mg/kg body weight) through gavage (Monday to Friday, once daily) for 6 weeks. At the end of the experiment, pancreatic tumor weights were recorded. Data represent the mean ± S.D. *, #, % = significantly different from control, P<0.05. Lower Panel, Body weight of tumor-bearing mice during the experiment. Data represent the mean ± S.D. (B), Effects of resveratrol on apoptosis and cell proliferation in tumor tissues. Immunohistochemistry was performed in tumor tissues isolated from control and resveratrol treated mice. Apoptosis was measured by TUNEL assay and cell proliferation was measured by Ki67 staining. Data represent the mean ± S.D. *, #, % = significantly different from control, P<0.05. (C), Effects of resveratrol on markers of apoptosis and cell cycle in tumor tissues. Western blot analyses were performed to examine the expression of Bim, caspase-3, PCNA, cell cycle inhibitors p27 and p21. The β-actin was used as a loading control. (D), Effects of resveratrol on ERK, PI3K/AKT and FOXO proteins isolated from tumor tissues. Western blot analyses were performed to examine the expression of p-ERK, ERK, p-PI3K (Tyr458), PI3K, p-AKT (Ser473), AKT, p-FOXO (Ser256), FOXO, p-FOXO3a (Ser253), and FOXO3a. The β-actin was used as a loading control.

We next validated our in vitro data that resveratrol inhibits growth of pancreatic cancer cells by inhibiting ERK and PI3K/AKT pathways leading to activation of FOXO transcription factors. PANC-1 cells were orthotopically implanted in Balb C nude mice and treated with resveratrol (0–60 mg/kg body weight) through gavage (Monday to Friday, once daily) for 6 weeks. As shown in Fig. 7A, resveratrol inhibited pancreatic tumor growth in Balb C nude mice in a dose-dependent manner. Furthermore, resveratrol has no effect on the body weight of tumor bearing mice, although mice gained weight during the treatment.

We next examined the effects of resveratrol on apoptosis and cell proliferation in tumor tissues derived from control and resveratrol treated mice (Fig. 7B). Resveratrol induced apoptosis and inhibited cell proliferation in tumor tissues in a dose-dependent manner. Since resveratrol induced apoptosis and inhibited cell proliferation, we next sought to examine the molecular mechanisms of the effects of resveratrol. Resveratrol induced the expression of Bim, p27/KIP1 and p21/CIP1, cleaved caspase-3, and inhibited the expression of PCNA (Fig. 7C). Since ERK and AKT phosphorylates FOXO, we next sought to examine the effects of resveratrol on the dephosphorylation/activation FOXOs. Western blot analyses were performed to examine the expression of p-ERK, ERK, p-PI3K (Tyr458), PI3K, p-AKT (Ser473), AKT, p-FOXO (Ser256), FOXO, p-FOXO3a (Ser253), and FOXO3a. As shown in Fig. 7D, resveratrol inhibited the phosphorylation of ERK, PI3K (Tyr458), p-AKT (Ser473), p-FOXO (Ser256) and p-FOXO3a (Ser253). It has no significant effect on the expression of total proteins. These data confirm our hypothesis that resveratrol activates FOXO transcription factors by inhibiting ERK and PI3K/AKT pathways in pancreatic cancer cells.

## Discussion

In this paper we have demonstrated, for the first time, that polyphenolic compound resveratrol causes growth arrest and induces apoptosis through activation of FOXO transcription factors. Resveratrol induces apoptosis through caspase-3 activation, and causes growth arrest through induction of p21 and p27 and inhibition of cyclin D1 which are the downstream targets of FOXO. Inhibition of PI3K/AKT and MEK/ERK pathways acts together to enhance the activation of FOXO transcriptional activity, and phosphorylation deficient triple mutants of FOXO further enhance resveratrol-induced FOXO activity and apoptosis. Inhibition of FOXO1, FOXO3a and FOXO4 by shRNA blocked resveratrol-induced cell cycle arrest and apoptosis. Resveratrol-induced suppression of PANC1 tumor growth in nude mice was associated with inhibition of ERK, PI3K, AKT, FOXO1 and FOXO3a phosphorylation, and induction of apoptosis in tumor tissues. Resveratrol also induced the expression of FOXO target genes such as Bim, p27/KIP1 and p21/CIP1 in pancreatic tumor tissues. Similarly, others have also demonstrated the antiproliferative and proapoptotic effects of resveratrol in pancreatic cancer [30], [31], and here we further added the role of FOXO transcription factors in mediating the effects of resveratrol. Since resveratrol is a nontoxic polyphenolic compound, it can be safely used for the treatment and/or prevention of pancreatic cancer.

Resveratrol exerts its anticancer and chemopreventive properties against various types of cancer [32]. Some of the beneficial effects of resveratrol may be due to its ability to suppress autophagy by targeting p70 S6 kinase (S6K1) [33]. Resveratrol suppresses proliferation of various types of cancer cells, including breast, lung, pancreas, liver, stomach, prostate, colon, leukemia, and medulloblastoma [7]. Antitumor activities of resveratrol have also been demonstrated in several mouse models of cancer [7], although the precise mechanisms and pathways involved remain unclear. In the present study, we have discovered a new mechanism by which resveratrol inhibits pancreatic tumor growth i.e. activation of FOXO transcription factors.

FOXO transcription factors have been shown to regulate tissue homeostasis in the pancreas, diabetes and cancer. In recent years, they have emerged as critical transcriptional integrators among pathways regulating proliferation, survival, differentiation and angiogenesis. FOXO regulates apoptotic and cell cycle genes such as Bim [28], Fas ligand [34], p27/KIP1 [28], and Bcl-6 [35]. Our results show that resveratrol induced the expression of PTEN and inhibited the phosphorylation/activation of AKT in PANC-1 cells. We also demonstrate that the inhibition of AKT phosphorylation is correlated with the activation of FOXO transcription factors and induction of Bim, p21^/CIP1^ and p27^/KIP1^. In vivo, resveratrol also induced the expression of FOXO target genes such as Bim, p27/KIP1 and p21/CIP1 in pancreatic tumor tissues. In other studies, we have demonstrated that anti-angiogenic effects of chemopreventive agents EGCG, resveratrol and sulforaphane are mediated through activation of FOXO transcription factors [25], [36]. Together, these studies suggest that activation of FOXO transcription factors by chemopreventive agents may regulate cell cycle, apoptosis and angiogenesis.

The PI3K/AKT and MAPK pathways can be activated by Kras [24], [25], [37]. AKT and ERK both have been shown to directly phosphorylate and inactivate FOXO transcription factors, resulting in cytoplasmic retention, inactivation, and inhibition of the expression of FOXO-regulated genes, which control various processes such as metabolism, cell cycle, cell death and oxidative stress [38]. Our results show that resveratrol inhibited cytoplasmic phosphorylation of FOXOs, and enhanced FOXO nuclear translocation, FOXO-DNA binding and transcriptional activities. The inhibition of PI3K/AKT and MEK/ERK pathways induced FOXO transcriptional activity and apoptosis. Overexpression of phosphorylation deficient triple mutants of FOXOs induced FOXO activity, and further enhanced resveratrol-induced FOXO activity and apoptosis, whereas deletion of FOXOs genes by shRNA abrogated resveratrol-induced cell cycle arrest and apoptosis. Resveratrol-induced suppression of tumor growth in mice was associated with inhibition of ERK and PI3K/AKT pathway and activation of FOXO1 and FOXO3a. Taken together, these studies demonstrate that the activation of FOXOs has significant implication for the treatment and prevention of pancreatic cancer, where Kras is activated in about 90% patients, and PI3K/AKT and MEK/ERK pathways are highly activated.

In addition to phosphorylation, the regulation of FOXO transcription factors by acetylation has also been demonstrated. The acetylation/deacetylation of FOXO can be regulated by p300, Cbp (CREB-binding protein) and Pcaf (p300/CBP-associated factors) in response to oxidative stress or DNA binding, followed by deacetylation by class I and II histone deacetylases [39], including Sirt1 [40]. Many of the SIRT1 substrates are transcription factors and key regulators described to take part in cancer development, such as the NFκB, the DNA repair factor Ku70, the tumor suppressor gene p53, and the FOXOs. The relationship between tumorigenesis and SIRT1 activity is still open to debate. Although some studies have suggested that SIRT1 may function as a tumor promoter because of its increased expression in some types of cancers, other studies have demonstrated that SIRT1 levels are reduced in some other types of cancers thereby acting as tumor suppressors. With an improved SIRT1 deacetylation assay, it has been reported that polyphenolic stimulators Epigallocatechin galate (EGCG), epicatechin galate (ECG) and myricetin stimulated SIRT1 under stabilizing conditions, whereas without stabilization, these polyphenols strongly inhibited SIRT1, probably due to H2O2 formation [41]. In the present study, resveratrol inhibited the expression of SIRT1, SIRT2 and SIRT3 in pancreatic cancer cells, thus suggesting a role of SIRT1 as a tumor suppressor in cancer cells.

However, further studies are needed to examine the consequences of acetylation/deacetylation of FOXO transcription factors on tumorigenesis and angiogenesis.

In summary, we have demonstrated that resveratrol induces cell cycle arrest and apoptosis through regulation of FOXO transcription factors. Pharmacological and genetic inhibitions of PI3K/AKT and MEK/ERK pathways can have synergistic effects on the activation of FOXO transcription factors through dephosphorylation and nuclear retention, resulting in regulation of FOXO-target genes. Our study provides important information regarding the mechanisms by which resveratrol regulates cell cycle, apoptosis and tumor growth through activation of FOXO transcription factors. Thus, our studies suggest that inhibition of ERK and AKT pathways act together to activate FOXO transcription factors which are involved in resveratrol-mediated pancreatic tumor growth suppression.

## Methods

### Ethics Statement

All experiments involving animals were approved by the Institutional Animal Care and Use Committee (IACUC) of the University of Texas Health Science Center at Tyler, protocol # 373.

### Reagents

Antibodies against PTEN, phospho-AKT, AKT, ras, phospho-ERK, ERK, phospho-p38, p38, p21/CIP1, p27/KIP1, cyclin D1, p-FOXO1, FOXO, pFOXO3a, FOXO3a, Bim, PCNA, caspase-3, and β-actin were purchased from Cell Signaling Technology, Inc. (Danvers, MA). Enhanced chemiluminescence (ECL) Western blot detection reagents were from Amersham Life Sciences Inc. (Arlington Heights, IL). Terminal Deoxynucleotidyl Transferase Biotin-dUTP Nick End Labeling (TUNEL) assay kit was purchased from EMD Biosciences/Calbiochem (San Diego, CA). Resveratrol was purchased from LKT Laboratories, Inc. (St. Paul, MN). Kits for Terminal Deoxynucleotidyl Transferase Biotin-dUTP Nick End Labeling (TUNEL) and caspase-3 assays were purchased from EMD Biosciences/Calbiochem (San Diego, CA).

### Cell Culture

PANC-1, MIA PaCa-2, AsPC-1 and Hs 766T cells were obtained from the American Type Culture Collection (Manassas, VA) and cultured in RPMI 1640 supplemented with 10% fetal bovine serum (FBS) and 1% antibiotic-antimycotic (Invitrogen) at 37°C in a humidified atmosphere of 95% air and 5% CO_2_.

### Transient Transfection

Cells were plated in 60-mm dishes in RPMI 1640 containing 10% FBS and 1% penicillin-streptomycin mixture at a density of 1×10^6^ cells/dish. The next day transfections were performed using LipofectAMINE (Invitrogen Life Technologies, Carlsbad, CA). Cells were transfected with expression constructs encoding wild type PTEN (pSG5L-HA-PTENwt), mutant PTEN (pSG5L-HA-PTEN-G 129E and pSGL5-HA-PTEN-G 129R), wild type-AKT (pUSE-WT-AKT), constitutively active-AKT (pUSE-CA-AKT), dominant negative-AKT (pUSE-DN-AKT), or the corresponding empty vectors (pSG5L or pUSE ) in the presence of an expression vector pCMV-LacZ (Invitrogen Life Technologies) expressing β-galactosidase. For each transfection, 2 µg of DNA was diluted into 50 µl of medium without serum. After the addition of 3 µl of LipofectAMINE into 50 µl Opti-MEM medium, the transfection mixture was incubated for 10 min at room temperature. Cells were washed with serum-free medium, the transfection mixture was added, and cultures were incubated for 24 hrs in the incubator. The next day, culture medium was replaced with fresh RPMI 1640 containing 10% FBS and 1% penicillin-streptomycin mixture and resveratrol was added for desired times for the measurement of apoptosis and protein expression.

### Western Blot Analysis

Western blots were performed as we described earlier [42]. In brief, cells were lysed in a buffer containing 10 mM Tris-HCl (pH 7.6), 150 mM NaCl, 0.5 mM EDTA, 1 mM EGTA, 1% SDS, 1 mM sodium orthovanadate, and a mixture of protease inhibitors (1 mM phenylmethylsulfonyl fluoride, 1 µg/ml pepstatin A, 2 µg/ml aprotinin). Lysates were sonicated for 10 s, centrifuged for 20 min at 10,000×g and stored at −70°C. Equal amounts of lysate proteins were run on 10% SDS-PAGE and electrophoretically transferred to nitrocellulose. Nitrocellulose blots were blocked with 6% nonfat dry milk in TBS buffer (20 mM Tris-HCl (pH 7.4), and 500 mM NaCl), and incubated with primary antibody in TBS containing 1% bovine serum albumin overnight at 4°C. Immunoblots were washed three times (15, 5 and 5 min each) with TBST (TBS and 0.01% Tween 20). Immunoreactivity was detected by sequential incubation with horseradish peroxidase-conjugated secondary antibody and ECL reagents.

### Measurement of Apoptosis

Cells were treated with resveratrol and apoptosis was measured by TUNEL assay as per manufacturer instructions (EMD Biosciences).

### Caspase-3 Assay

Cells (3×10^4^ per well) were seeded in a 96-well plate with 200 µl culture medium. Approximately 16 h later, cells were treated with various doses of resveratrol to induce apoptosis. Casapse-3 activity was measured by a fluorometer as per manufacturer's instructions (EMD Biosciences).

### Cell Cycle Analysis

PANC-1 cells were transfected with either scrambled or FKHR/FKHRL1/AFX-shRNA (FOXO-shRNA) plasmids. FKHR/FKHRL1/AFX-shRNA (FOXO-shRNA) plasmids have been described elsewhere [28]. Transfected cells were treated with resveratrol (10 µM) for 24 h. Cell cycle analysis was performed as we described elsewhere [28].

### Clonogenic Assay

Pancreatic cancer cells (10^4^ cells) were mixed with 0.3% agarose and layered over a solid base of 0.5% agarose in RPMI in the absence or presence of resveratrol in 33-mm dishes. The cultures were incubated in humidified 5% CO2, 95% air at 37°C for 21 days and then stained with the nitro blue tetrazolium. Colonies of >120 µm in diameter were counted using a microscope.

### AKT Enzyme Assays

Cells were lysed in 1 ml of lysis buffer (50 mM Tris-HCl, pH 7.5, 150 mM NaCl, 1% (w/v) Triton X-100, 10% glycerol, 1 mM EDTA, 1 mM dithiothreitol, 1 mM benzamidine, 1 mM phenylmethylsulfonyl fluoride, 1 mg/ml bacitracin, 1 mM Na_3_VO_4_, 30 mM NaPP_i_, 10 mM NaF, 100 nM okadaic acid). Following centrifugation, the supernatants were incubated for 2 h at 4 °C with protein A-Sepharose beads coated with 2.5 µl of anti-Akt antibody. Immunoprecipitates were washed three times with the lysis buffer and twice with the kinase assay buffer (50 mM Tris-HCl, pH 7.5, 10 mM MgCl_2_, 1 mM dithiothreitol) and assayed using GSK-3 peptide (GRPRTSSFAEG) as substrate as described [43].

### Evaluation of mRNA expression levels by quantitative Real Time-PCR

For the quantification of gene amplification, Real-time PCR was performed using an ABI 7300 Sequence Detection System in the presence of SYBR- Green. Briefly, RNA isolated with or without treatment with the chromatin modifiers using TRIzol (Life Technologies) were reverse transcribed. cDNA reactions were amplified with QPCR SYBR Green Mix (Applied Biosystems) and the following gene-specific primers:

Sirt1 (5′-tgt ggt aga gct tgc att ga-3′, 5′- gcc tgt tgc tct cct cat ta-3′)

Sirt2 (5′-aag act gtc aga gcc tgg tg-3′, 5′- ata atc atc ccc agg aaa gg-3′)

Sirt3 (5′-tgg aat tgg tga cct agg aa-3′, 5′-acc tgt aat ccc agt tac cc-3′)

HK-GAPD (5′-gag tca acg gat ttg gtc gt-3′, 5′-ttg att ttg gag gga tct cg-3′)

Target sequences were amplified using incubated at 95°C for 10 min, followed by 40 cycles of 95°C for 15 s and 60°C for 1 min. HK-GAPD is used as endogenous normalization control. All assays were performed in triplicate and were calculated on the basis of ΔΔ*C*t method.

### Luciferase Assay

Pancreatic cancer cell lines were transfected with empty vector, FOXO1-TM, FOXO3a-TM or FOXO4-TM along with reporter plasmids, p6xDBE-luc and pRL-TK [44]. The FOXO expression vectors (wild type and phosphorylation deficient mutants) and FOXO-luciferase constructs have been described elsewhere [44]. After 24 h, transfection medium was replaced with culture medium and cells were treated with resveratrol (0–20 µM). After incubation of 24 h, the relative luciferase activity, i.e. firefly enzyme activity divided by that of the Renilla enzyme, was determined using Dual Luciferase Reporter Assay System (Promega, Madison, WI) according to the manufacturer's protocol.

### Electrophorectic mobility shift assay (EMSA)

FOXO probes were end-labeled with [γ-^32^P] dATP by incubating oligodeoxyribonucleotide strands with 5× reaction buffer and 10 U T4 polynucleotide kinase for 1 h at 37°C. Then labeled oligonucleotides were allowed to anneal at room temperature for 10 min and 20 µg protein from each sample was used in 25 µl binding reactions, which consisted of 1 µg poly dI-dC, in 5× binding buffer (50 mM Tris HCl; pH 8.0, 750 mM KCl, 2.5 mM EDTA, 0.5% Triton-X 100, 62.5% glycerol (v/v) and 1 mM DTT). To determine specificity of DNA binding, samples were incubated with or without 20 ng of unlabeled competitor DNA for 10 min at room temperature. Then 0.1 ng of labeled probe was added and samples were further incubated for 20 min at room temperature. Samples were separated on a 5% non-denaturing polyacrylamide gel in 0.5% TBE and visualized by autoradiography.

### Antitumor activity of resveratrol

PANC-1 (0.2×10^6^ cells mixed with Matrigel, Becton Dickinson, Bedford, MA) cells in a final volume of 0.1 ml were injected into the pancreas of Balb/c *nu/nu* mice (4–6 weeks old). The mice were purchased from the National Cancer Institute, Frederick, MD. After one week, mice (7 mice per group) were treated with resveratrol (0, 20, 40 and 60 mg/kg body weight) through gavage (Monday to Friday, 5 days a week for 6 weeks, once daily). At the end of the experiment, mice were euthanized as per approved protocol (The University of Texas Health Science Center at Tyler, Texas), and tumors were isolated and weighed.

### Immunohistochemistry

Imunohistochemistry of pancreatic cancer cells, and tumor tissues collected on week 6 was performed as we described elsewhere [42]. TUNEL assays were performed as per manufacturer's instructions (Roche Applied Sciences).

### Statistical Analysis

The mean and SD were calculated for each experimental group. Differences between groups were analyzed by one or two way ANOVA, followed by Bonferoni's multiple comparison tests using PRISM statistical analysis software (GrafPad Software, Inc., San Diego, CA). Significant differences among groups were calculated at P<0.05.
